# Identification of a natural recombinant transmissible gastroenteritis virus between Purdue and Miller clusters in China

**DOI:** 10.1038/emi.2017.62

**Published:** 2017-08-23

**Authors:** Xin Zhang, Yunnuan Zhu, Xiangdong Zhu, Hongyan Shi, Jianfei Chen, Da Shi, Jing Yuan, Liyan Cao, Jianbo Liu, Hui Dong, Zhaoyang Jing, Jialin Zhang, Xiaobo Wang, Li Feng

**Affiliations:** 1 Division of Swine Infectious Diseases, State Key Laboratory of Veterinary Biotechnology, Harbin Veterinary Research Institute of the Chinese Academy of Agricultural Sciences, Harbin 150069, China; 2 Molecular Biology (Gembloux Agro-Bio Tech), University of Liège (ULg), Liège 5030, Belgium

**Keywords:** identification, natural recombinant, transmissible gastroenteritis virus

## Abstract

Transmissible gastroenteritis virus (TGEV) is an infective coronavirus (CoV) that causes diarrhea-related morbidity and mortality in piglets. For the first time, a natural recombination strain of a TGEV Anhui Hefei (AHHF) virus between the Purdue and the Miller clusters was isolated from the small intestine content of piglets in China. A phylogenetic tree based on a complete genome sequence placed the TGEV AHHF strain between the Purdue and the Miller clusters. The results of a computational analysis of recombination showed that the TGEV AHHF strain is a natural recombinant strain between these clusters. Two breakpoints located in the open reading frame 1a (ORF1a) and spike (S) genes were identified. The pathogenicity of the TGEV AHHF strain was evaluated in piglets, and the results show that TGEV AHHF is an enteric pathogenic strain. These results provide valuable information about the recombination and evolution of CoVs and will facilitate future investigations of the molecular pathogenesis of TGEV.

## INTRODUCTION

Coronaviruses (CoVs) include four genera (alpha-, beta-, gamma- and delta-CoVs) and are the main cause of global outbreaks of porcine diarrhea, which result in substantial economic losses.^1^ CoV members that cause diarrhea in pigs have been identified;^2^ transmissible gastroenteritis virus (TGEV), porcine epidemic diarrhea virus (PEDV) and porcine respiratory virus (PRCV, a natural TGEV deletion mutant) are alpha-CoVs;^3, 4, 5^ porcine hemagglutinating encephalomyelitis virus is a beta-CoV;^6^ and porcine deltacoronavirus (PDCoV) is a delta-CoV.^2, 7^ CoVs have high-mutation rates due to recombination, resulting in changes in tissue tropism, transmission route and host specificity.^2^

TGEV is the etiological agent of transmissible gastroenteritis (TGE), which can cause severe diarrhea with high morbidity in suckling piglets.^8^ TGEV is an enveloped virus with a positive-sense RNA genome of 28.5 kb that was first reported in the United States in 1946.^9^ TGEV is currently divided into two distinct genogroups: the Miller cluster and the Purdue cluster.^10, 11, 12, 13, 14^ The genome of TGEV contains a leader sequence at the 5′ end and a poly (A) tail at the 3′ end, and it encodes four structural proteins, including spike (S), envelope (E) and membrane (M) proteins and a nucleoprotein (N), and five non-structural proteins, including replicases 1a (pp1a) and 1ab (pp1ab) and proteins 3a, 3b and 7.

In this study, a natural recombinant strain of TGEV AHHF between the Purdue and Miller clusters was isolated from the small intestine content of piglets in China. The results provide valuable information about CoV recombination and evolution and will facilitate future investigations into the molecular pathogenesis of TGEV.

## MATERIALS AND METHODS

### Specimen collection

In 2015, tissue from one small intestine and 14 anal tissue strips were harvested from a pig farm in Anhui Hefei (AHHF). The clinical symptoms were vomiting, yellow and watery diarrhea, rapid weight loss and dehydration. Intestinal content was collected from an infected piglet and was mixed with PBS at a ratio of 1:5. The suspension was then centrifuged at 6000*g* for 15 min at 4 °C and was filtered using a 0.22-μm filter.

### Virus passaging and titration

The TGEV AHHF strain was passaged in PK15 cells containing 0.2 μg/mL trypsin. The virus was collected at 72 h post-infection. The AHHF strain was passaged in PK15 cells for 10 generations. PK15 cells were plated in 96-well plates for 50% tissue culture infective dose (TCID_50_) assays. The TCID_50_ was determined using a method described by Reed and Muench.^15^

### Reverse transcription (RT)-PCR and DNA sequencing

Total RNA was extracted from PK15 cells infected with TGEV AHHF using a QIAamp Viral RNA Mini kit (Qiagen, Germany) following the manufacturer’s instructions. Viral complementary DNA (cDNA) was generated via reverse transcription using PrimeScript reverse transcriptase (TaKaRa, Dalian, China) according to the manufacturer’s instructions. Primers were designed as shown in Supplementary Table S1. The 5′ and 3′ ends of the viral genome were amplified using the SMART RACE cDNA Amplification Kit (Clontech, Mountain View, CA, USA). Each PCR amplicon was obtained via one-step RT-PCR. All PCR reactions were performed as follows: pre-denaturation for 5 min at 94 °C; 30 cycles of denaturation for 30 s at 95 °C, annealing for 30 s at 55 °C, and extension for 2.5 min at 72 °C; followed by a final 10-min total extension step at 72 °C. Amplified fragment lengths of each PCR amplicon are shown in Supplementary Table S1.

### Sequence analysis

Sequences of the TGEV reference strains were obtained from GenBank (Supplementary Table S2). Nucleotide sequences and predicted amino acid sequences were analyzed with DNASTAR software using the CLUSTAL W program (DNAstar Inc., Madison, WI, USA). Phylogenetic trees were constructed with MEGA5.1 software using the neighbor-joining (NJ) method. Bootstrap values were estimated for 1000 replicates.

### Western blotting

Western blotting was performed as previously described.^15^ Briefly, cell lysates of TGEV AHHF-infected PK15 cells were separated via 12% PAGE and were transferred to a nitrocellulose membrane. Cell lysates of TGEV-uninfected PK15 cells were used as a control. After blotting, the membrane was incubated with mAb 5E8^16^ (1:1000) for 1 h to detect the N protein of TGEV AHHF. The membrane was washed three times with PBST and was then inoculated with DyLight 800-labeled mouse IgG antibodies (H+L) (1:10 000, KPL, USA) for 45 min. Images were visualized using an Odyssey Infrared Imaging System (LI-COR).

### Indirect immunofluorescence assay

PK15 cells were infected with the TGEV AHHF strain for 36 h and were fixed with paraformaldehyde (4%). The cells were blocked with 5% skim milk and were incubated with mAb 5E8 as described previously.^16^ Briefly, the cells were incubated with mAb 5E8^16^ (1:100) for 1 h to detect the N protein of TGEV AHHF for 60 min; afterwards, they were washed three times with PBST. Subsequently, the cells were incubated with FITC-labeled goat anti-mouse IgG (1:100, KPL, USA). The stained cells were washed three times with PBST and were subsequently examined under a fluorescence microscope.

### Immunohistochemistry

Immunohistochemistry (IHC) detection was performed as described previously.^17^ MAb 5E8 was used to visualize the N protein of TGEV AHHF. Briefly, the slides were incubated with mAb 5E8 (1:100) at 4 °C overnight. Subsequently, the slides were incubated with HRP-labeled goat anti-mouse IgG (1:2000, Sigma) for 1 h. The slides were visualized using the 3,3′-diaminobenzidine liquid substrate system.

### Electron microscopy

TGEV AHHF was purified via differential centrifugation. First, a cell culture of TGEV AHHF was centrifuged at 6000*g* for 30 min at 4 °C and then at 80 000*g* for 2 h at 4 °C. After ultracentrifugation, samples were dyed with 2% phosphotungstic acid (pH 7.0–7.4) and were adsorbed in a 300-mesh copper net for 2 min. The samples were observed via electron microscopy after negative staining.

### Real-time PCR

Tissue samples of the same weight were excised from TGEV-inoculated piglets and were ground up. Total RNA was extracted using a Spin Column RNA Purification Kit (Qiagen) according to the manufacturer’s protocols. Specific primers for amplifying the N gene of TGEV were used (F: 5′-TTT TGT TTG GAA GCT ATT GGA CT-3′ R2: 5′-CCT TTG GCA AGT GGT ATT TGT G-3′). The probe used was 5′-FAM-CGT GAC TTC TAT CTG GTC GCC ATC TTC-TAMRA-3′. Quantitative real-time RT-PCR was performed using a LightCycler 480 II (Roche Life Science, South San Francisco, CA, USA) in a total volume of 20 μL. The PrimeScript RT reagent kit with gDNA Eraser (Perfect Real Time, TaKaRa) was used for RT-PCR according to the manufacturer’s protocol. Melting curves were obtained using pEGFP-TGEV-N^16^ as a standard. Quantitative analysis of the data was performed using an absolute quantification study model. The real-time RT-PCR analysis provided linear curves for 10^1^–10^8^ copies/μL of TGEV standards.

### Recombination analysis

Recombination analysis was completed using RDP4 software.^18^ Methods, including RDP, Bootscan and SiScan, were used. Likely parental isolates and recombination breakpoints were detected under default settings. Criteria for determining recombination and breakpoints were a *P*-value <10^−6^ or a recombination score >0.6.

### Animal challenge

Three specific pathogen-free piglets were challenged with 10^6^ TCID_50_ of TGEV AHHF, while three other specific pathogen-free piglets were challenged with DMEM as a control. Experimental animals were infected orally. After 96 h of inoculation, all of the experimental animals were killed, and the samples were collected. The Animal Ethics Committee approval number is Heilongjiang-SYXK-2006-032.

## RESULTS

### Isolation and characterization of the TGEV AHHF strain

**Figure 1:**
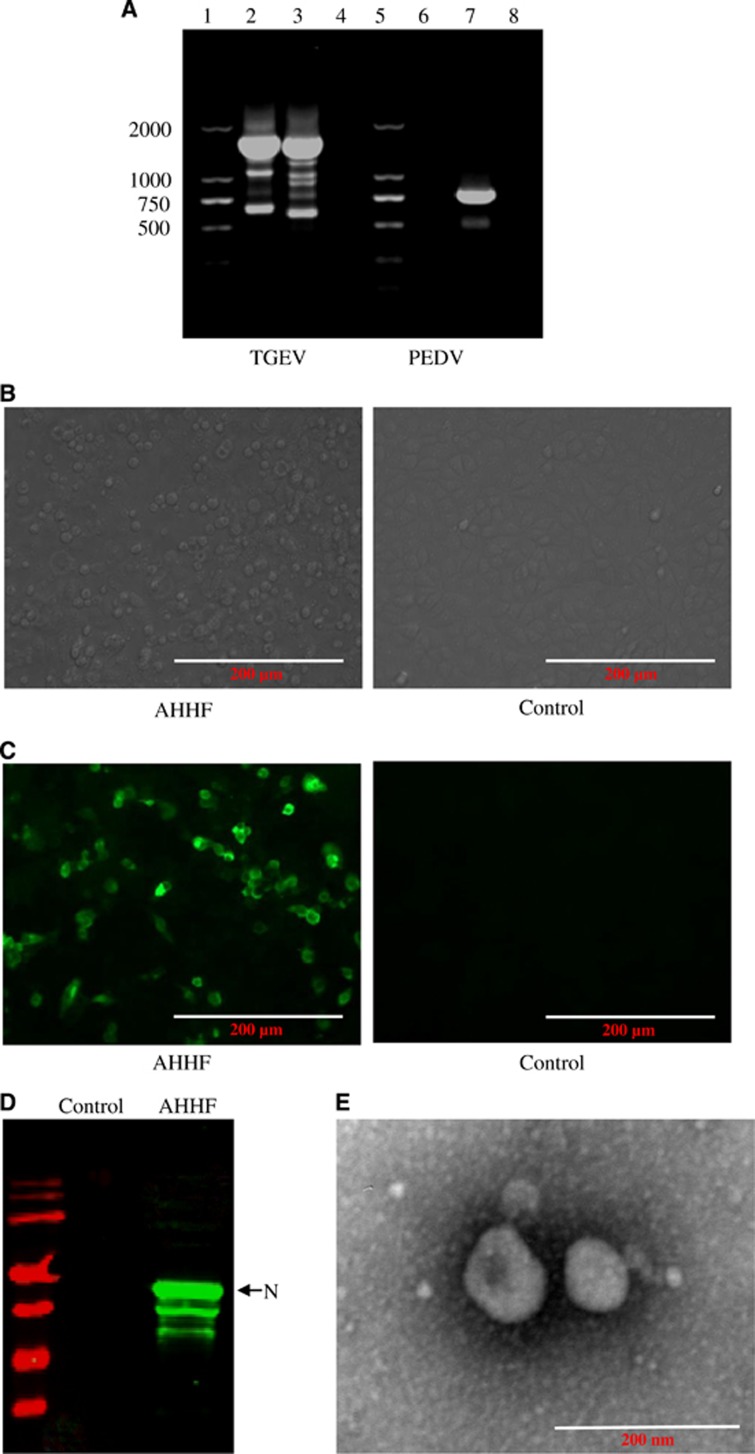
Isolation and characterization of the TGEV AHHF strain. (**A**) PCR amplification of TGEV AHHF. Lanes 1, 5: DL marker 2000. Lanes 2, 6: cDNA of TGEV AHHF. Lane 3: cDNA of TGEV attenuated H. Lanes 4, 8: negative control. Lane 7: cDNA of PEDV CV777. (**B**) Cytopathic effects in TGEV AHHF-infected PK15 cells. (**C**) IFA identification of TGEV AHHF. (**D**) Western blot identification of TGEV AHHF. (**E**) Electron microscopy observation of TGEV AHHF.

Tissue from one small intestine and 14 anal strips were harvested from a pig farm in AHHF in China. Vomiting and diarrhea were present in the piglets at this farm. TGEV detection showed positive results in the small intestine tissue but negative results in the anal strips. The detection rate of TGEV was 6.67%. PEDV was negative in all harvested samples. To determine the causative agent of the diarrhea that was present at the AHHF pig farm in China, total RNA isolated from the small intestine content of the pigs was extracted and subjected to RT-PCR. The results show that the sample was positive for TGEV but negative for PEDV (Figure 1A). After passaging in the PK15 cell line, cytopathic effects were found, as evidenced by the round shape and increase in size of the cells, the formation of syncytia, and the detachment of the cells from the plate (Figure 1B). Western blotting using mAb to the N protein of 5E8^16^ showed that the N protein was present in TGEV AHHF-infected PK15 cells but not in the negative control cells (Figure 1C). The results of the IFA show specific fluorescence in the TGEV AHHF-infected PK15 cells but not in the negative control cells (Figure 1D). When analyzed via electron microscopy, typical coronavirus-like particles with diameters of 100–120 nm were observed (Figure 1E). The virus was named the TGEV AHHF strain. The TCID_50_ of TGEV AHHF (10^5.12^/mL) was measured using the Reed–Muench method.

### Complete genome sequence of TGEV AHHF

**Figure 2:**
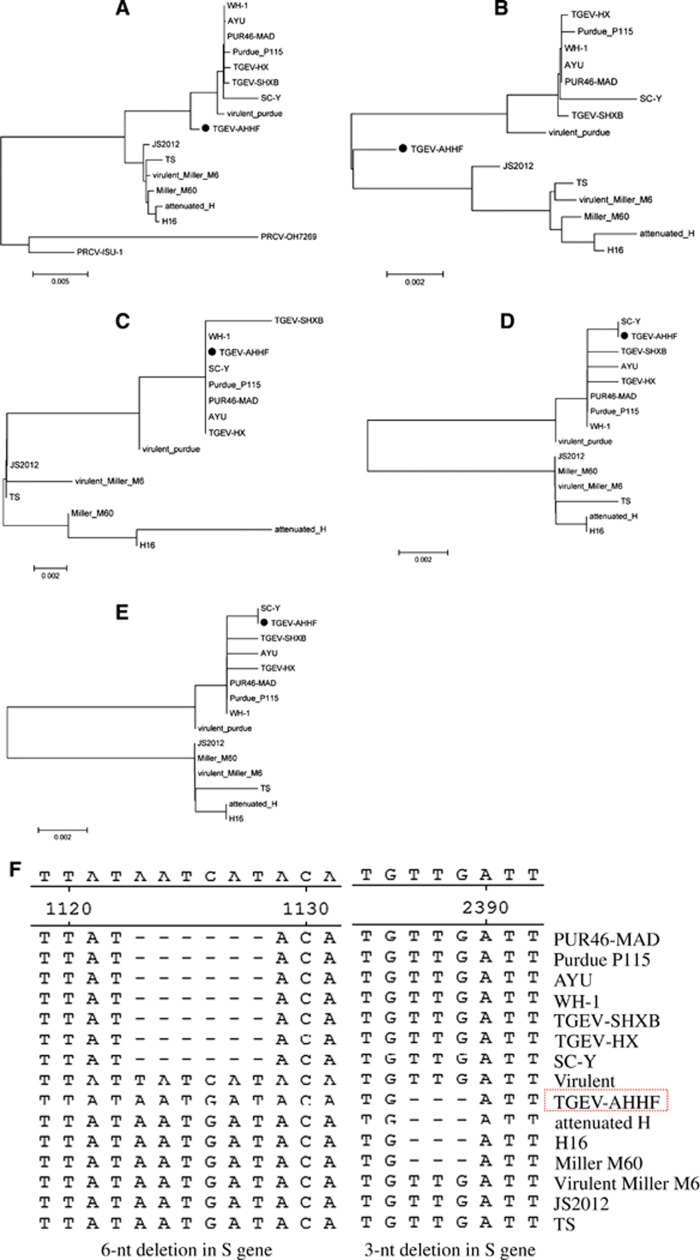
Phylogenetic analysis of TGEV AHHF based on the complete genome sequence and the genes encoding structural proteins. (**A**) Complete genome. (**B**) S gene. (**C**) E gene. (**D**) M gene. (**E**) N gene. (**F**) 6-nt deletion and 3-nt deletion in the S gene of TGEV.

The complete genome sequence of the TGEV AHHF strain was deduced from overlapping cDNA fragments as previously described.^19^ Furthermore, sequencing was performed for the field sample before tissue cultures. The sequence of the field sample of TGEV AHHF was consistent with that of the PK15 cell culture passages. The sequence was submitted to GenBank under accession number KX499468. A total of 28 614 nt (containing poly(A)) were determined for the TGEV AHHF strain, encompassing the 5′ untranslated region (UTR) (nt 1–314), ORFs 1a (nt 315–12 368), 1b (nt 12 329–20 368), S (nt 20 365–24 708), 3a (nt 24 827–25 042), 3b (nt 25 139–25 873), E (nt 25 860–26 108), M (nt 26 119–26 907), N (nt 26 920–28 068), 7 (nt 28 074–28 310) and the 3′ UTR (nt 28 311–28 614). The 5′ UTR included a potential short AUG-initiated ORF (nt 114–121) beginning within a Kozak sequence (5′-UCUAUGAA-3′). The 3′ end of the genome contained a 273-nt untranslated sequence and a poly (A) tail. Upstream of the poly (A) tail was a 5′-GGAAGAGC-3′ octameric sequence. The complete genome sequence of the AHHF strain was compared with those of other TGEV reference strains and the PRCV-ISU-1 strain. Phylogenetic analysis showed that the AHHF strain was more closely related to the Purdue strain group than to the Miller strain group (Figure 2A).

### Genetic features of the structural proteins encoded by the TGEV AHHF genome

The nucleotide sequence of the TGEV AHHF strain S gene was 4 347 nt in length, encoding a predicted S protein containing 1448 amino acids (aa) (Table 1). The S protein of AHHF had the same length as those of attenuated H, H16, and Miller M60 (Table 1). The TGEV AHHF strain E (249 nt), M (789 nt), and N (1149 nt) genes encoded predicted proteins containing 82 aa, 262 aa, and 382 aa, respectively (Table 1). Phylogenetic analysis of the complete TGEV AHHF S gene showed that it was closely related to the Miller strain group (Figure 2B). However, phylogenetic analysis of E (Figure 2C), M (Figure 2D) and N (Figure 2E) showed that they were closely related to the Purdue strain group.

Previous research has shown that there are two deletions in the TGEV S protein (a 3-nt deletion and a 6-nt deletion).^12, 13^ A 3-nt deletion was found at nt 2387–2389 in the TGEV AHHF S gene, and this deletion has also been found in the attenuated strains H, H16 and Miller M60 (Figure 2F). However, a 6-nt deletion in the S gene at nt 1123–1128 was not found in the AHHF strain but was found in the S gene of the WH-1, AYU, PUR46-MAD, TGEV-HX, SC-Y, TGEV-SHXB and Purdue P115 strains. Sequence analysis revealed no deletions or insertions in the E, M, and N genes of the TGEV AHHF strain.

### Genetic features of the non-structural proteins encoded by the TGEV AHHF genome

**Figure 3:**
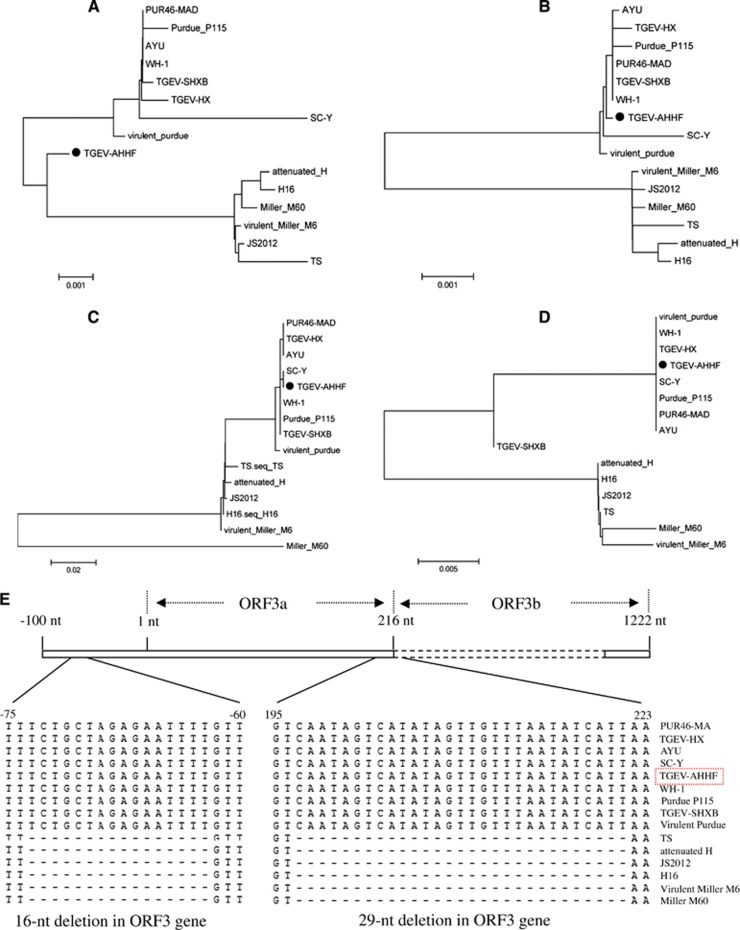
Phylogenetic analysis of TGEV AHHF based on genes encoding non-structural proteins. (**A**) ORF1a. (**B**) ORF1b. (**C**) ORF3. (**D**) ORF7. (**E**) 16- and 29-nt deletions in the ORF3 gene of TGEV.

The ORF1a gene of TGEV AHHF was predicted to encode a 4017-aa pp1a protein, while the ORF1b gene was predicted to encode a 2680-aa pp1b protein (Table 1). The nucleotide sequences of the TGEV AHHF strain ORF3a and ORF3b genes were 219 and 735 nt in length, respectively, and they encoded predicted ORF3a and ORF3b proteins containing 71 aa and 244 aa, respectively. The predicted ORF7 was 237 nt in length, encoding a protein containing 78 aa. Nucleotide sequence analysis indicated that there were no deletions or insertions in the ORF1a, ORF1b, or ORF7 genes of the TGEV AHHF (Table 1). Phylogenetic analysis of the ORF1a genes suggested that the TGEV AHHF strain was more closely related to the Miller strain group (Figure 3A). However, phylogenetic analysis of the ORF1b (Figure 3B), ORF3 (Figure 3C) and ORF7 (Figure 3D) genes suggested that they shared a close relationship with the Purdue strain group.

Previous research had demonstrated the existence of two deletions in the TGEV ORF3a gene: a 16-nt deletion before the initiation codon ‘ATG’ and a 29-nt deletion at the stop codon ‘TAA’.^13^ As shown in Figure 3E, such a 16-nt deletion before the initiation codon ‘ATG’ and a 29-nt deletion at the stop codon ‘TAA’ were found in all strains of the Miller cluster (based on the phylogenetic analysis of the ORF3 gene), including TS, attenuated H, JS2012, H16, virulent Miller M6 and Miller M60. However, no deletions or insertions were found in the ORF3 gene of the AHHF strain (Figure 3E).

### Recombination of TGEV AHHF

**Figure 4:**
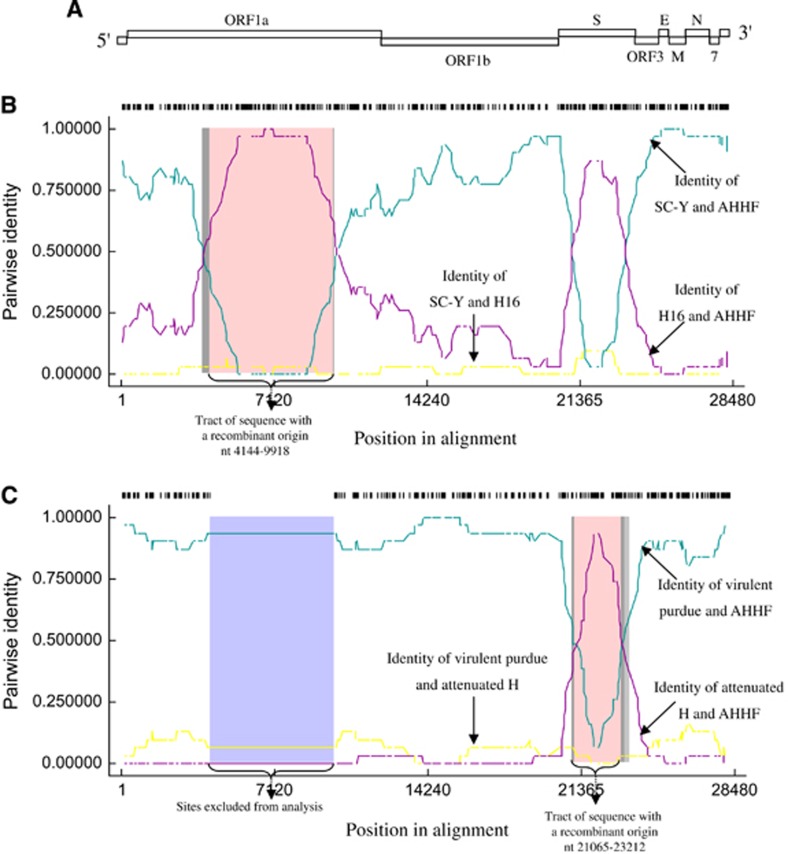
Recombination analysis of TGEV AHHF with other TGEV strains. (**A**) TGEV genome organization. (**B**) Recombination of SC-Y and H16 located at nt 4 144–9 918 of the ORF1a gene. (**C**) Recombination of virulent Purdue and attenuated H located at nt 21 065–23 212 of the S gene.

To further analyze the association between TGEV AHHF and the existing isolates, a genetic analysis was completed using RDP4 software.^18^ TGEV genome organization is shown in Figure 4A. Breakpoints for potential recombination zones were found at two locations, the ORF1a gene (nt 4144–9918 [aa 1286–3210 in pp1a]) (Figure 4B) and the S gene (nt 21 065–23 212 [aa 222–938 in the S protein]) (Figure 4C). The major parent strain of the recombination located in the ORF1a gene was SC-Y, and the minor parent strain was H16 (Figure 4B). SC-Y of TGEV (accession No. DQ443743) was isolated from Sichuan Qionglai in China.^20^ H16 of TGEV (accession No. FJ755618)^19^ was isolated from Shanghai in China. Both SC-Y and H16 viruses show a strong pathogenicity in piglets. The major parent strain of the recombination located at the S gene was virulent Purdue, and the minor parent strain was attenuated H (Figure 4C). These results indicate that TGEV AHHF was the result of a natural recombination event between the Miller cluster and the Purdue cluster.

### Analysis of the stem-loop structures at the breakpoint sites

The predicted stem-loop structures of TGEV AHHF, SC-Y, H16, virulent Purdue and attenuated H strains were determined using MaxExpect RNA structure software (version 5.8.1) (Web Servers for RNA Secondary Structure Prediction, http://rna.urmc.rochester.edu/RNAstructure.html). At nt positions 4084–4143 (upstream of the 4144–9918 breakpoint), the RNA structure of TGEV AHHF was similar to that of SC-Y (Supplementary Figure S1A). Furthermore, at nucleotide positions 9993–10 052 (downstream of the 4144–9918 breakpoint), we found that the RNA structure of TGEV AHHF was similar to that of SC-Y (Supplementary Figure S1B). This finding verified that the reorganization located at the S gene of TGEV AHHF was from strains SC-Y and H16. At nucleotide position 21 001–21 064 (upstream of the 21 065–23 212 breakpoint), the RNA structure of TGEV AHHF was similar to that of the virulent Purdue strains (Supplementary Figure S1C). Furthermore, at nucleotide position 23 213–23 272 (downstream of the 21 065–23 212 breakpoint), the RNA structure of TGEV AHHF was similar to that of the virulent Purdue strains (Supplementary Figure S1D). This result also verified that the reorganization located at the S gene of TGEV AHHF was from the virulent Purdue and attenuated H strains.

### Clinical signs in TGEV AHHF-infected piglets

To evaluate the pathogenicity of TGEV AHHF in piglets, three piglets were orally inoculated with 10^6^ TCID_50_ of TGEV AHHF. Three other piglets were inoculated with DMEM. Vomiting and diarrhea were observed 96 h post-inoculation (P.I.). None of the piglets inoculated with TGEV AHHF died within the 96 h P.I. period. The control piglets did not show vomiting or diarrhea.

### Histopathological manifestations in the TGEV AHHF-infected piglets

**Figure 5:**
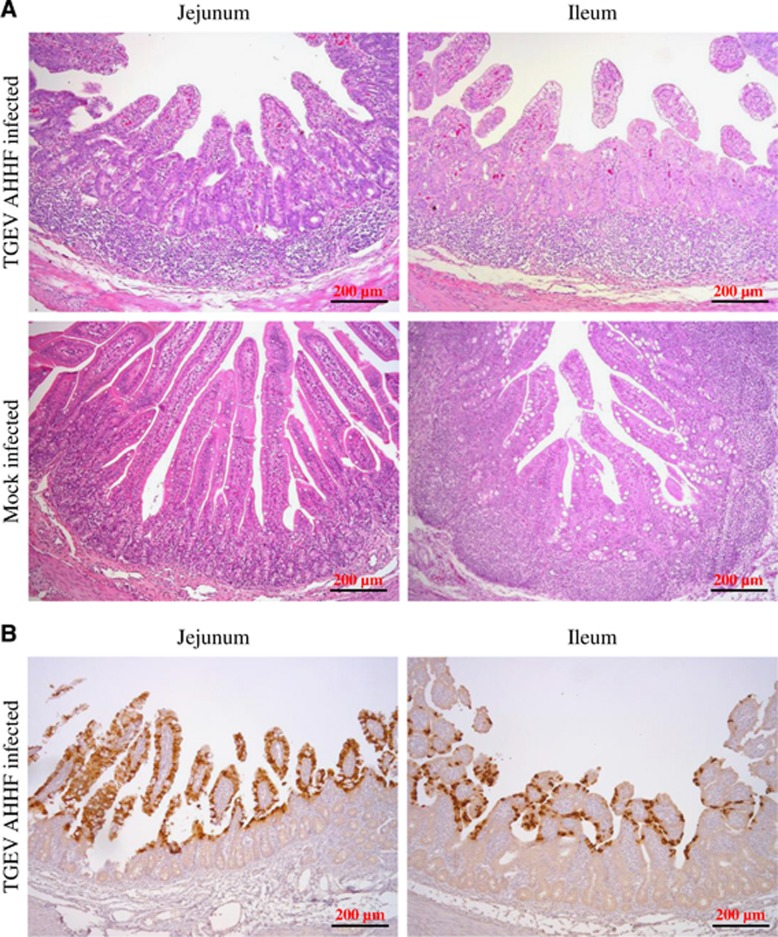
Pathological changes and IHC of TGEV AHHF-inoculated piglets. (**A**) Pathological changes in tissues collected from the jejunum and ileum of TGEV-inoculated piglets. (**B**) IHC assay using a mAb in the jejunum and ileum of TGEV-inoculated piglets.

All piglets were sacrificed at 96 h P.I. Pathological changes were mainly observed in the jejunum and ileum. Figure 5A shows the symptoms of villous atrophy, lymphoid aggregation of lymphocytes, and vacuolar degeneration of mucosal epithelial cells in both the jejunum and the ileum tissues of the TGEV-infected piglets but not in those of the mock-infected piglets. Furthermore, IHC was utilized to demonstrate the location of TGEV AHHF using mAb 5E8 (specific to the N protein of TGEV). As shown in Figure 5B, the N protein of TGEV AHHF was recognized by mAb 5E8 in jejunum and ileum tissues from piglets inoculated with TGEV AHHF. Most labeling for TGEV AHHF was observed in the villi of the jejunum and ileum.

### Viral distribution in TGEV AHHF-infected piglets

**Figure 6:**
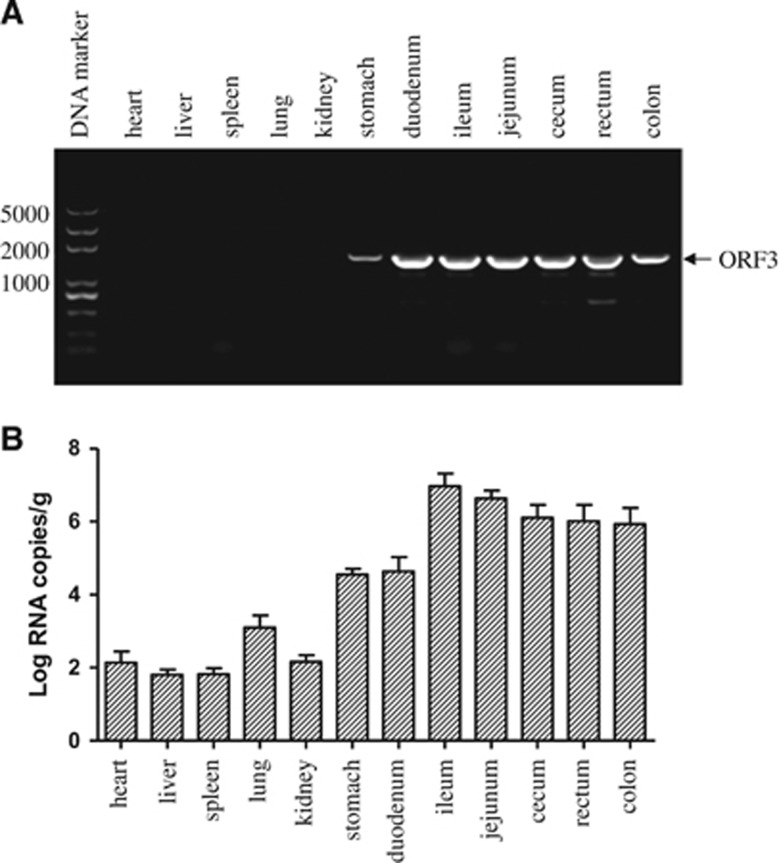
The PCR results and viral RNA distribution of piglet tissues inoculated with TGEV AHHF. (**A**) The PCR results from piglet tissues inoculated with TGV AHHF. (**B**) Quantification of viral RNA in piglet tissues via real-time PCR.

TGEV AHHF was detected in all samples of the TGEV AHHF-infected piglets. Figure 6A shows that TGEV AHHF was detected in all samples of infected stomachs and intestines. The viral RNA levels at 96 h P.I. and the copies/g of TGEV RNA reached the highest levels (7–8 log_10_) in the jejunum (Figure 6B). At 96 h P.I., the viral levels in the stomach reached 4–5 log_10_ RNA copies/g of TGEV RNA. The amount of virus in the parenchymal organs (heart, liver, spleen, lung and kidney) was relatively low (1–3 log_10_ RNA copies/g).

## DISCUSSION

TGEV is an excellent model for understanding the pathogenesis and evolution of CoV.^21, 22, 23^ In 2015, intestinal tissue of a pig with AHHF-induced diarrhea was collected from a pig farm China, and the infection was accompanied by vomiting and diarrhea in piglets. The TGEV AHHF strain was successfully harvested from PK15 cells, after which, we sequenced the entire genome of this strain. Whole-genome analysis showed that TGEV AHHF was a natural recombination strain between the Purdue and the Miller clusters.

The 5′ and 3′ UTRs of CoVs are critically important for viral replication and transcription.^23^ The ‘slippery’ heptanucleotide sequence of 5′-TTTAAAC-3′ and a pseudoknot structure are both critical for viral RNA synthesis and are involved in ribosomal frame shifting.^24^ In this study, there were no deletions or insertions in the 5′ and 3′ UTRs of the AHHF strain. Furthermore, the slippery sequence and pseudoknot structure were both found in TGEV AHHF, suggesting that the replication and transcription mechanisms of TGEV AHHF were unchanged.

CoVs attach to their host cells via S interactions between the glycoprotein and the host cell receptor aminopeptide N (pAPN).^25^ A domain of the S protein encoded by nt 1518–2184 is efficiently recognized by pAPN.^26^ In the S gene, nt 217A, 385A, 646T, 655G or 665T greatly affects the enteric tropism of TGEV.^27, 28^ In this study, these positions (nt 217A, 385A, 646T, 655G or 665T) were unchanged in TGEV AHHF, suggesting that the AHHF strain is an enteric virus. The CoV S protein is the major immunogenic protein of the virus and can stimulate the body to produce antibodies with neutralizing activity.^29^ A mutation (T to G at nucleotide 1753) causing a serine (S) to an alanine (A) mutation at aa 585, which is located in the major antigenic sites A/B, influences the antigenicity of the TGEV S protein.^10^ Analyzing the S protein, we found that aa 585 is A, which can also be found in the attenuated TGEV H, H16, Miller M60, PUR46-MAD, Purdue P115, SC-Y and WH-1 strains but not in the HN2002, Miller M6, TS or virulent Purdue strains, indicating that the antigenicity of the S protein of AHHF may be changed. The neutralizing ability of TGEV AHHF relative to both the Purdue cluster and the Miller cluster should be evaluated in a future study.

A 6-nt deletion (nt 1123–1128) in the S gene has been considered a trait of the virus strains of the TGEV Purdue cluster.^30^ There is a 6-nt deletion in some Purdue cluster strains, including PUR-46 MAD and Purdue P115, but not in virulent Purdue.^31^ Purdue P115 is attenuated; therefore, this 6-nt deletion may play a role in the attenuation of TGEV.^28, 31^ In this study, the 6-nt deletion was not found in TGEV AHHF, suggesting that TGEV AHHF may be a virulent strain. This hypothesis was confirmed in the animal experiment, in which TGEV AHHF was used to inoculate piglets, which demonstrated that TGEV AHHF is pathogenic to piglets. Furthermore, a 3-nt deletion (nt 2386–2388) in the S gene that results in the deletion of aa 796V was found in the TGEV AHHF strain; this type of deletion was previously found in strains H16, attenuated H and Miller M60. The deletion of aa 796V may not influence the virulence of TGEV, as this deletion was found in both the H16 (virulent) strain and the attenuated H strain.

Previous studies have shown the potential involvement of the TGEV ORF3 gene in viral virulence or tropism.^1, 32^ Sequence analysis of the TGEV strains also showed large deletions and insertions (16 and 29 nt, respectively) in the ORF3a gene region.^33^ There are some uncertainties regarding the pathogenicity of the ORF3a deletion. Some researchers have found that the deletion in the ORF3a gene has a limited effect on virulence.^34^ Two large deletions in the ORF3 gene were not found in TGEV AHHF or in the Purdue cluster but were found in the strains of the Miller cluster. The effects of ORF3 on TGEV AHHF pathogenicity and tissue macrophages require further study using reverse genetics.

Recombination occurs among CoVs,^35, 36^ such as in PEDV^37, 38^ and canine CoV.^39^ Mixed infections under field conditions give rise to recombination events, and a new swine CoV generated by the recombination of TGEV and PEDV was recently identified.^40^ Regarding CoVs, homologous RNA recombination is one of the major ‘powers’ of genetic evolution and diversity.^41, 42^ Under field conditions, mixed infections are required to give rise to recombination events.^43^ Some TGEV viruses belonging to the Miller group have been found in China (attenuated H and TS),^14, 44^ and Purdue strains, such as TGEV HX and SHXB, are also prevalent in China,^12, 13^ providing increased opportunities for reorganization. The ORF1a and S genes are two important recombination sites (breakpoints). The breakpoint located in ORF1a has previously been found, for example, in human coronavirus OC43.^45^ Recombination breakpoints have also been identified in the S gene, for example, in human coronavirus NL63.^46^ In this study, two breakpoints, which are located in the ORF1a and S genes, were found, and will provide valuable information on CoV recombination and evolution.

The exact mechanism of recombination in CoVs is still unclear. During the replication process, a group of subgenomic RNAs (sgmRNAs) is generated,^23^ which increases the homologous recombination rate of closely related genes in different lineages of CoVs via template switching.^35^ The recombination site (breakpoint) in the CoV genome seems to be random, as different recombination viral strains have different breakpoints.^35^ It is very likely that CoV recombination occurs during genome transcription in the viral life cycle.

TGEV AHHF is a recombinant virus between the Miller cluster and the Purdue cluster. The immunogenicity of TGEV AHHF and the protection of piglets against viral attack from the Miller cluster or the Purdue cluster should be investigated. The capability of currently used vaccines to protect against TGEV AHHF attack also needs to be further assessed.

In summary, in this study, the first example of a natural recombination event in TGEV between the Purdue cluster and the Miller cluster was observed in China. Typical clinical signs, pathologic alterations and histological changes associated with TGE were observed in piglets challenged with the TGEV AHHF strain. Collectively, these results demonstrate that the TGEV AHHF strain is virulent and is transmissible in piglets. These results provide valuable information about CoV recombination and evolution and will facilitate future investigations of the molecular pathogenesis of TGEV.

## Figures and Tables

**Table 1:** Length of amino acids in the predicted structural and nonstructural proteins of TGEV AHHF strain compared with other TGEV strains

	**AHHF**	**WH-1**	**AYU**	**PUR46-MAD**	**HX**	**SC-Y**	**SHXB**	**Purdue P115**	**Virulent purdue**	**Attenuated H**	**H16**	**TS**	**Virulent Miller M6**	**Miller M60**	**JS2012**
Replicase 1a	4017	4017	4017	4017	4017	4017	4017	4017	4017	4017	4017	4017	4017	4017	4017
Replicase 1b	2680	2680	2680	2680	2680	2680	2680	2680	2680	2680	2680	2680	2680	2680	2680
S	1448	1447	1447	1447	1447	1447	1447	1447	1449	1448	1448	1449	1449	1448	1449
ORF3a	71	71	71	71	71	71	71	71	71	72	72	72	72	72	72
ORF3b	244	244	244	244	244	244	244	244	244	244	244	244	244	–	244
E	82	82	82	82	82	82	82	82	82	82	82	82	82	82	82
M	262	262	262	262	262	262	262	262	262	262	262	262	262	262	262
N	382	382	382	382	382	382	382	382	382	382	382	382	382	382	384
ORF7	78	78	78	78	78	78	78	78	78	78	78	78	78	78	78
