# Supplementary material for: Identification of a natural recombinant transmissible gastroenteritis virus between Purdue and Miller clusters in China
Source: Emerg Microbes Infect. 2017 Aug 23;6(8):e74–. doi: 10.1038/emi.2017.62 (PMC5583670; doi:10.1038/emi.2017.62)
Supplement: Supplementary Table S1 [file emi201762x1.docx]

Supplementary Table S1. Primers used in this study.

| **Primer** | **Sequence** | **Position** | **Product size** |
| --- | --- | --- | --- |
| TGE-ORF1-0F1 | TCCGCCCTATTTCGTAAGT | 206-225 | 663 |
| TGE-ORF1-0F2 | CAGTTTGGCAATTTATGAGG | 849-868 | 1241 |
| TGE-ORF1-1F1 | ATTGAATTTGAAGGAGAGGAG | 735-755 |  |
| TGE-ORF1-1F2 | TCACCAACAATGACGATATAAC | 1954-1975 | 1822 |
| TGE-ORF1-2F1 | AAATGCTTTGTGCTTGGTGC | 1740-1759 |  |
| TGE-ORF1-2F2 | TTCCGTTGAGAGTCGTTGTC | 3542-3561 | 1907 |
| TGE-ORF1-3F1 | CTGTTGAAGAAAATCCTGAG | 3325-3344 |  |
| TGE-ORF1-3F2 | TGGCTCACCTTTCTTTACTC | 5212-5231 | 2129 |
| TGE-ORF1-4F1 | TTAAAGGCACCGTTGTCATC | 4858-4877 |  |
| TGE-ORF1-4F2 | AAGGTCTTCAAGCAAATCACTG | 6965-6986 | 1806 |
| TGE-ORF1-5F1 | GCAAATGTTAGAAATGCCTGTG | 6900-6921 |  |
| TGE-ORF1-5F2 | AGCAGCAGATTCAAAGTTACC | 8685-8705 | 2373 |
| TGE-ORF1-6F1 | GTTTTCCTCTATGACTCACTCC | 8607-8628 |  |
| TGE-ORF1-6F2 | GTATGGTGGTGTTCTCAAAGT | 10960-10980 | 1719 |
| TGE-ORF1-7F1 | ACACTTGTGACCTTAGCGAACT | 10927-10948 |  |
| TGE-ORF1-7F2 | CTACGTGCAACATTACCGAACT | 12624-12645 | 2028 |
| TGE-ORF1-8F1 | GATTCTGGTGCTGTTGCTGAG | 12569-12589 |  |
| TGE-ORF1-8F2 | GTAGCACACAACTCCATCATC | 14576-14596 | 1667 |
| TGE-ORF1-9F1 | GTTATCGTAGTAGCAGCATTG | 14490-14510 |  |
| TGE-ORF1-9F2 | CGCATTGGTGTTATTAGGC | 16138-16156 | 2066 |
| TGE-ORF1-10F1 | TGTTCTCATGCGGCTGTAG | 16022-16040 |  |
| TGE-ORF1-10F2 | CGAGTGCGAGTATCAAATC | 18069-18087 | 2431 |
| TGE-ORF1-11F1 | TGGTAATCCAAAAGGCATTC | 17884-17903 |  |
| TGE-ORF1-11F2 | AATGAGCAACTTACCCTTCC | 20295-20314 | 1255 |
| TGE-S-1F1 | AAGGAAGGGTAAGTTGCTCA | 20293-20312 |  |
| TGE-S-1F2 | GGTCCATCAGTTACGCCGAA | 21529-21548 | 1299 |
| TGE-S-2F1 | GTGCCACAGTGTTTTCATTG | 21428-21447 |  |
| TGE-S-2F2 | GCAGTGCCACGAGTCCTATC | 22708-22727 | 2330 |
| TGE-S-3F1 | TAACGCCATGTGATGTAATC | 22559-22578 |  |
| TGE-S-3F2 | GCACAATCAAGTTCGTCAAG | 24869-24888 | 1245 |
| TGEV-ORF3-1F1 | TATTGAAAAAGTGCACGTCC | 24687-24708 |  |
| TGEV-ORF3-1F2 | CAACAGGAACCAGAAAATGA | 25912-25931 | 1131 |
| TGEV-M-F1 | CATCATTTTCTGGTTCCTGTT | 25910-25930 |  |
| TGEV-M-F2 | GGAGGGTTATGGGGTTGAAG | 27021-27040 | 1360 |
| TGEV-N-F1 | GCATTACCCAGCAGGAC | 26698-26704 |  |
| TGEV-N-F2 | AATAAATACAGCATGGAGGAGGAC | 28035-28058 | 483 |
| TGEV-ORF7-F1 | TCAGAAGTGGCAAAAGAACAG | 27913-27933 |  |
| TGEV-ORF7-F2 | ATTACACGTGCTTACCATTC | 28376-38395 |  |
| TGE-ORF3-F1 | CTGCTATTTTGCTGTTGT | 24583-24600 | 1602 |
| TGE-ORF3-F2 | ATCAGACGCTGTGCTATT | 26167-26184 |  |
