# Supplementary material for: Identification of a natural recombinant transmissible gastroenteritis virus between Purdue and Miller clusters in China
Source: Emerg Microbes Infect. 2017 Aug 23;6(8):e74–. doi: 10.1038/emi.2017.62 (PMC5583670; doi:10.1038/emi.2017.62)
Supplement: Supplementary Table S2 [file emi201762x2.docx]

Supplementary Table 2. Sequences used in this study.

| **Isolate** | **Accession no.** | **Origin** | **Collection date** | **Sort (Cluster)** |
| --- | --- | --- | --- | --- |
| H16 | FJ755618.2 | CHN | 2010 | Miller |
| Attenuated H | EU074218.2 | CHN | 2010 | Miller |
| Miller M6 | DQ811785.1 | USA | 2009 | Miller |
| Miller M60 | DQ811789 | USA | 2011 | Miller |
| TS | DQ201447 | CHN | 2006 | Miller |
| JS2012 | KT696544.1 | CHN | 2012 | Miller |
| AYU | HM776941.1 | CHN | 2012 | Purdue |
| SC-Y | DQ443743.1 | CHN | 2006 | Purdue |
| AYU | HM776941.1 | CHN | 2012 | Purdue |
| TGEV-HX | KC962433.1 | CHN | 2012 | Purdue |
| WH-1 | HQ462571 | CHN | 2011 | Purdue |
| TGEV-SHXB | KP202848.1 | CHN | 2013 | Purdue |
| Virulent purdue | DQ811787.1 | USA | 2009 | Purdue |
| PUR46-MAD | AJ271965.2 | USA | 2005 | Purdue |
| PRCV ISU-1 | DQ811787.1 | USA | 2009 | None |
| PRCV OH7269 | KR270796.1 | USA | 2014 | None |
