# Supplementary material for: Identification of a natural recombinant transmissible gastroenteritis virus between Purdue and Miller clusters in China
Source: Emerg Microbes Infect. 2017 Aug 23;6(8):e74–. doi: 10.1038/emi.2017.62 (PMC5583670; doi:10.1038/emi.2017.62)

**Supplementary Figure S1**

nt 4143

nt 4084

A


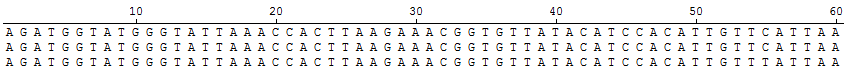

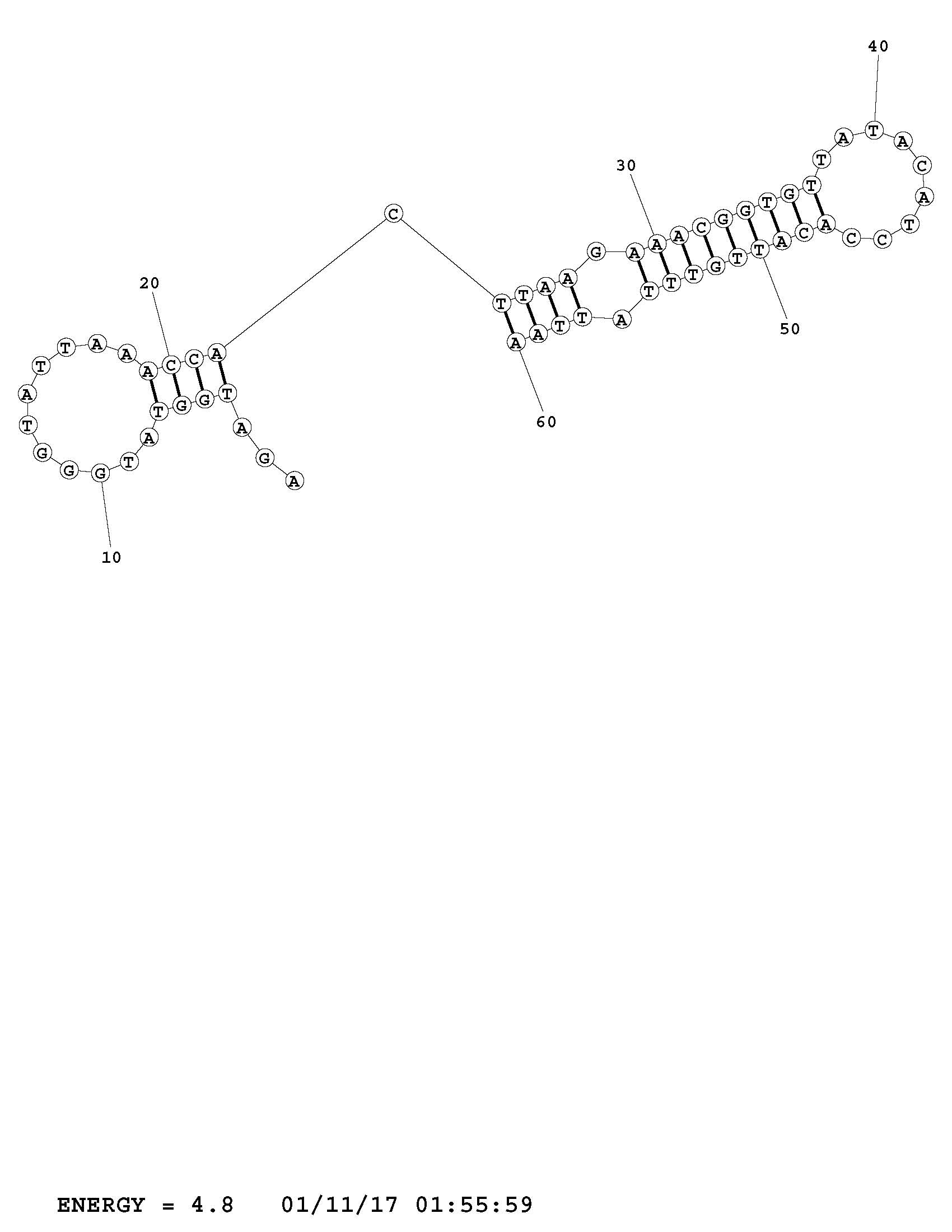

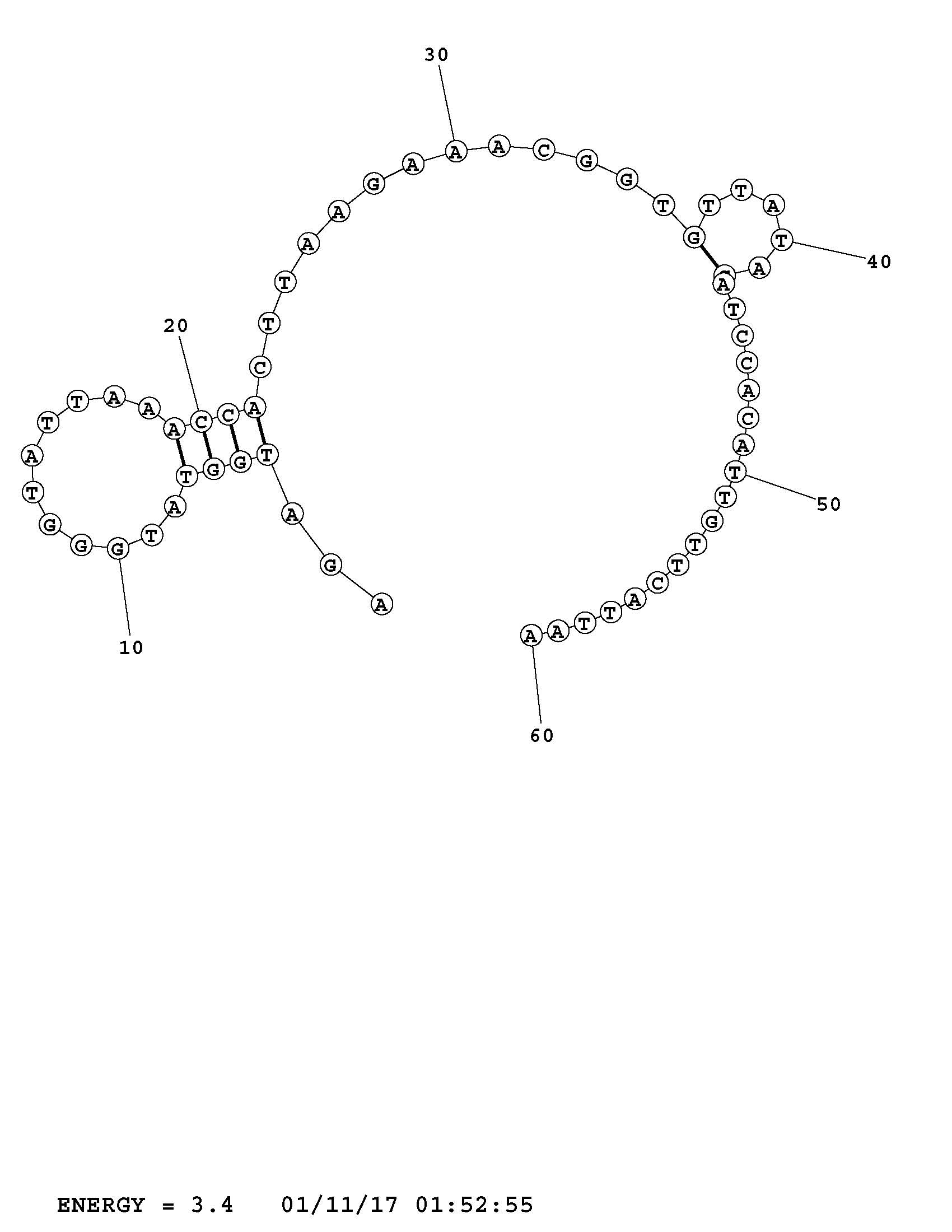

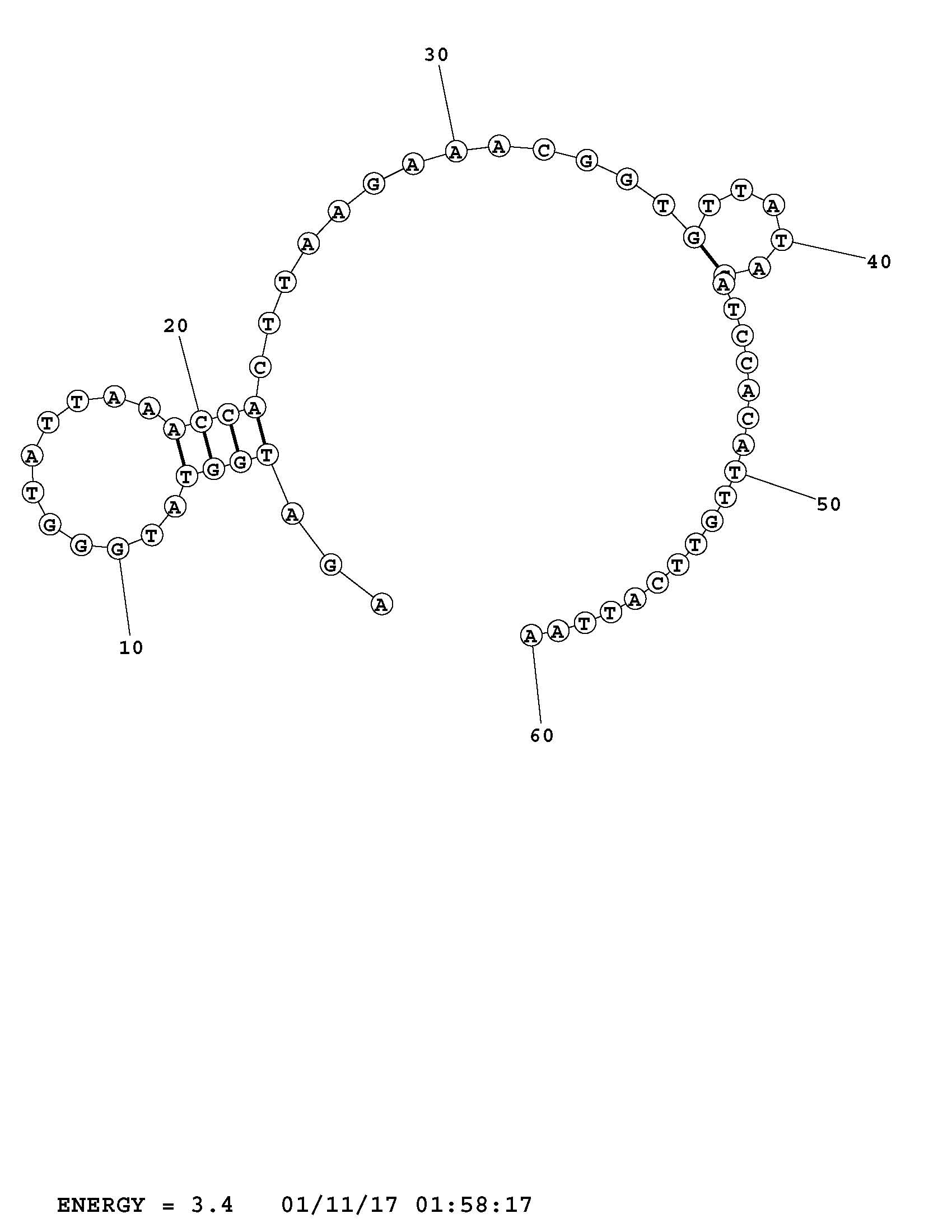


AHHF

SC-Y

H16

AHHF

SC-Y

H16


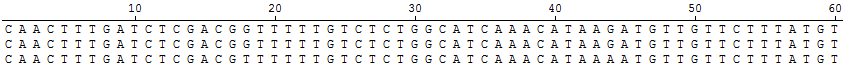


nt 10052

nt 9993


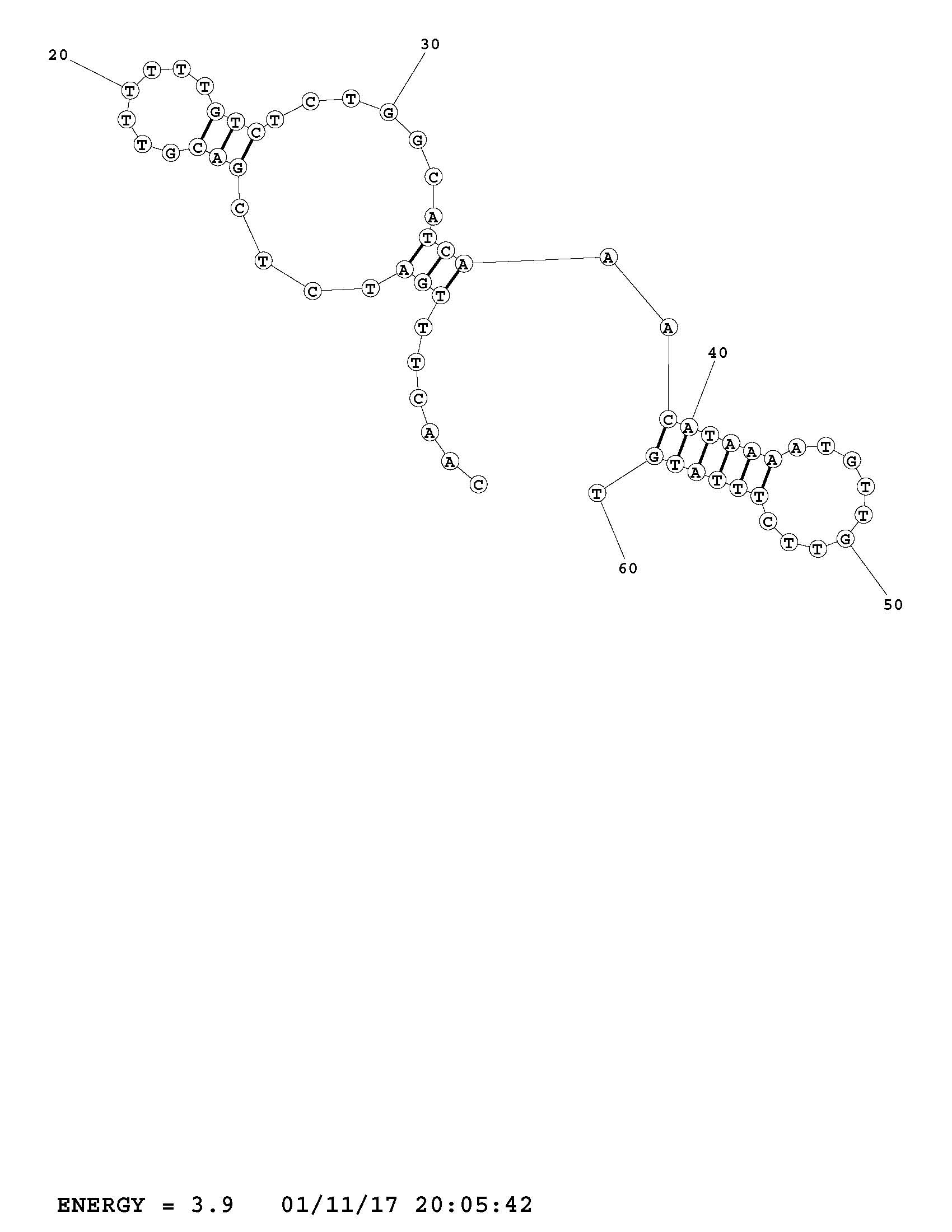

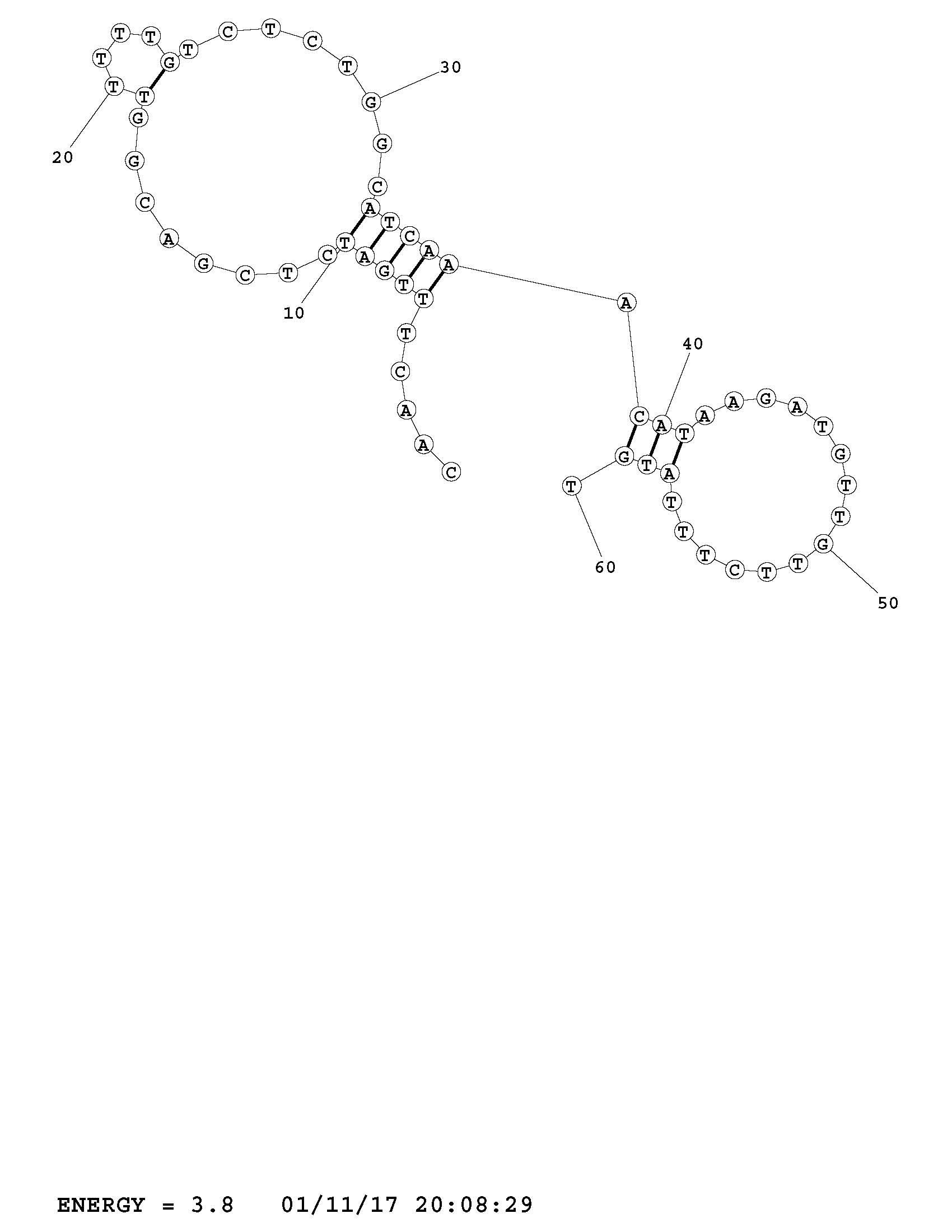

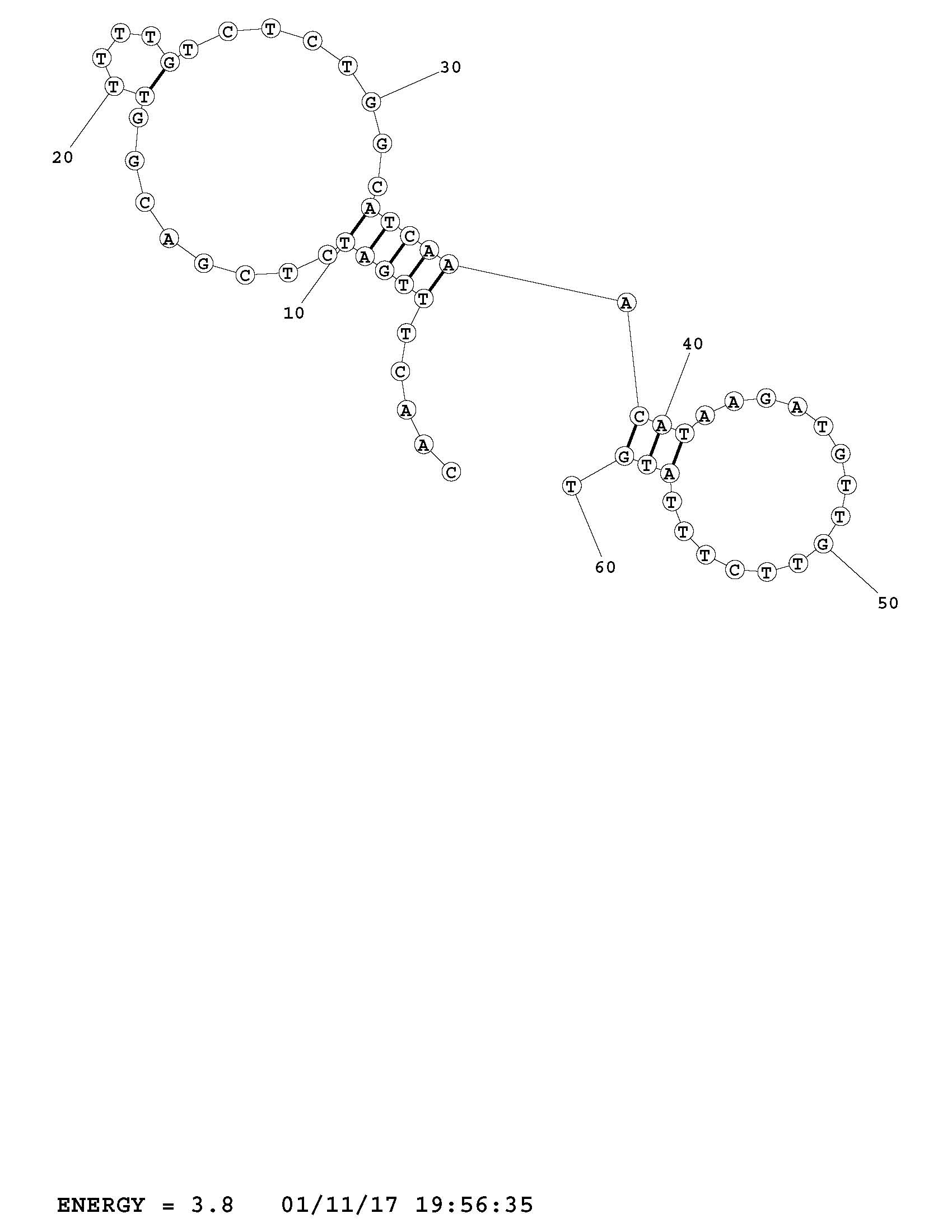


B

C

nt 21064

nt 21001


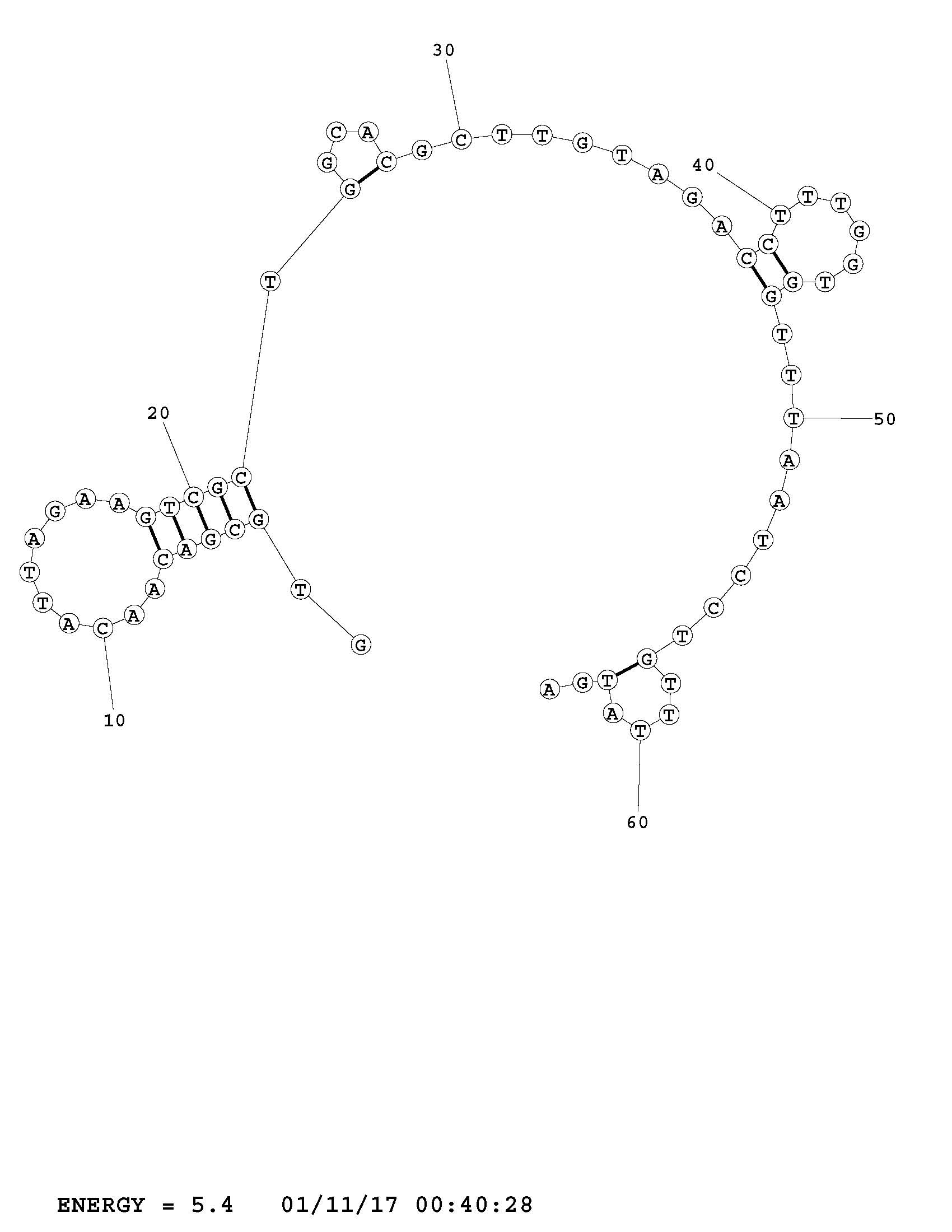

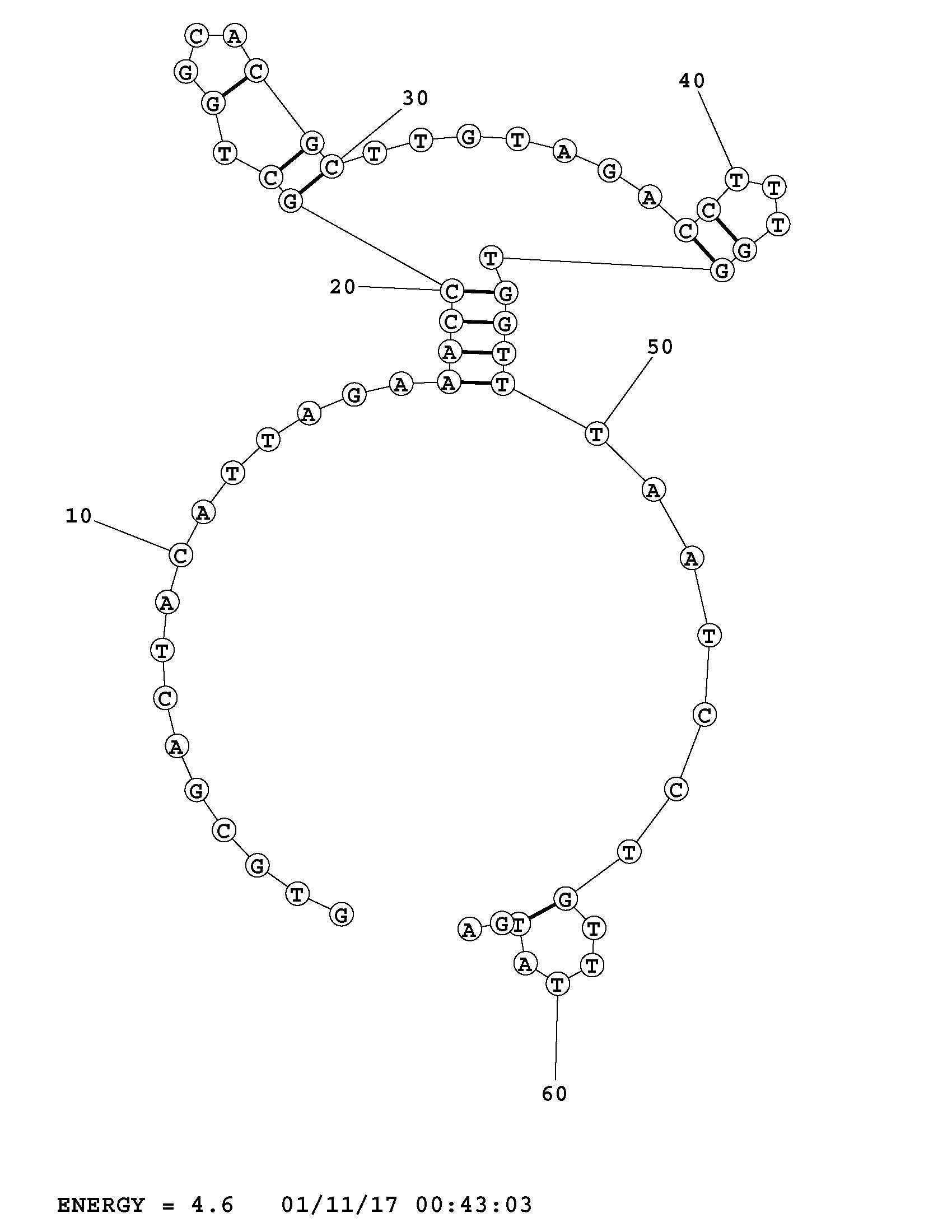

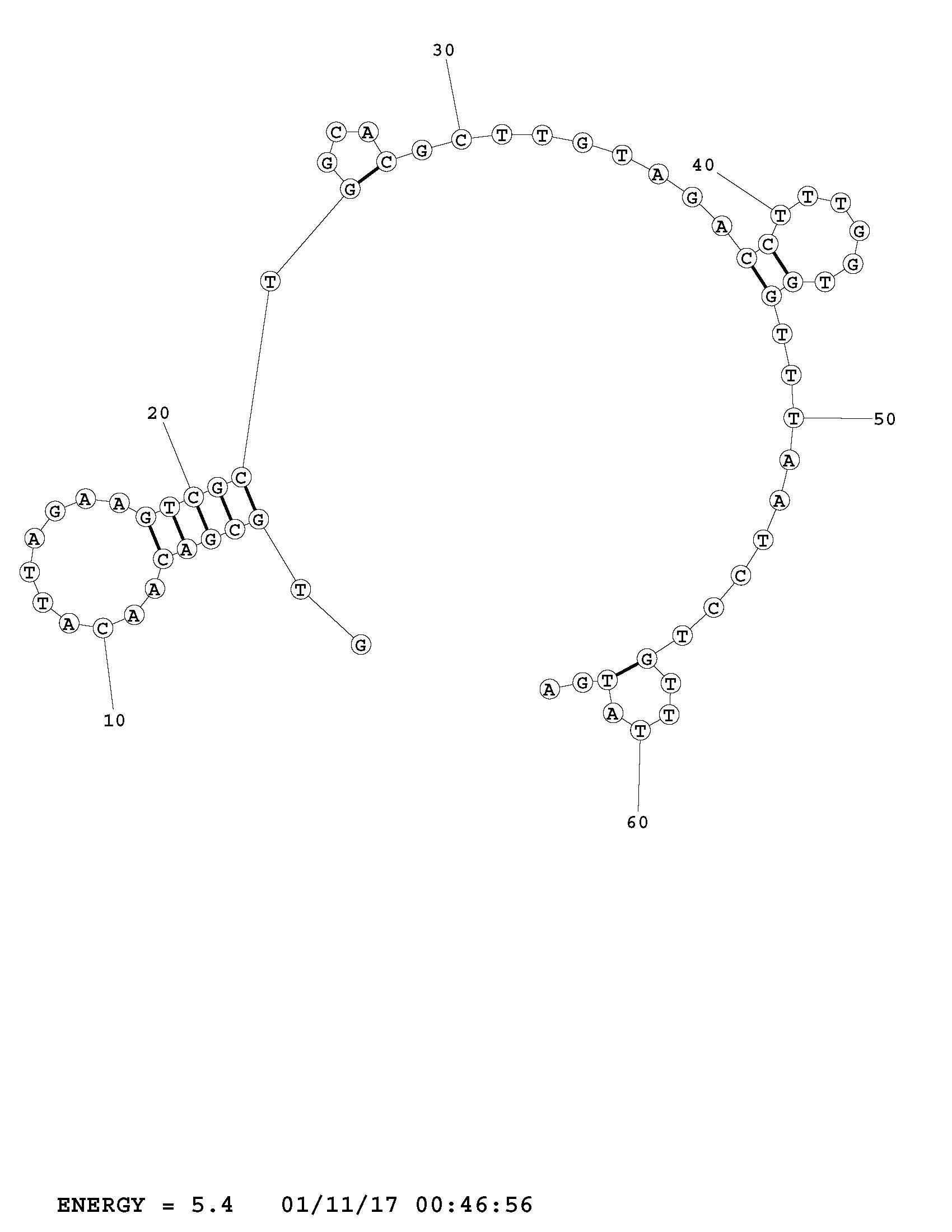


AHHF

Virulent purdue

Attenuated H


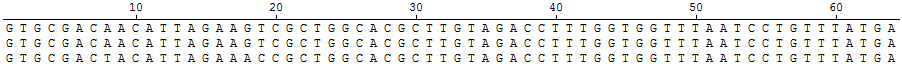


D

nt 23272

nt 23213


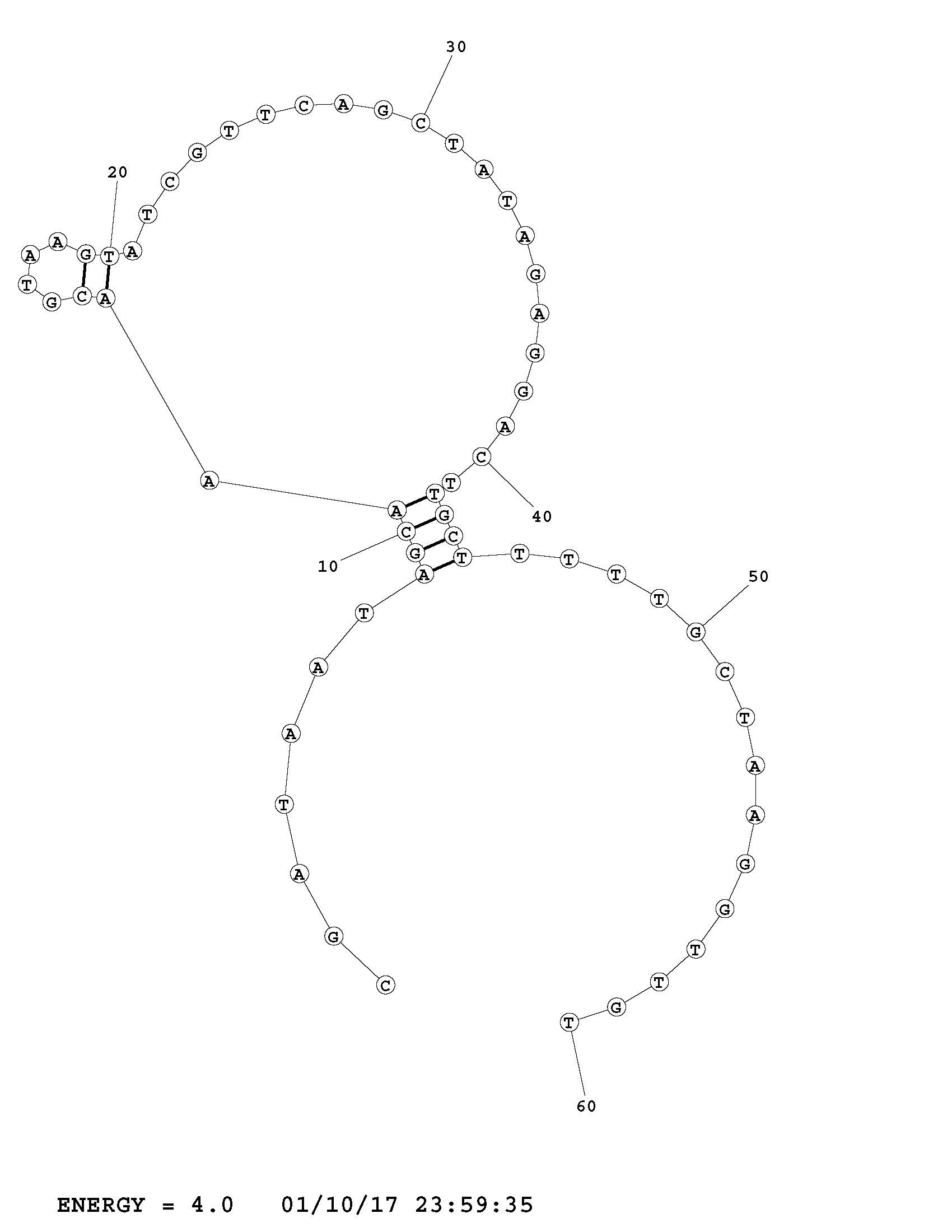

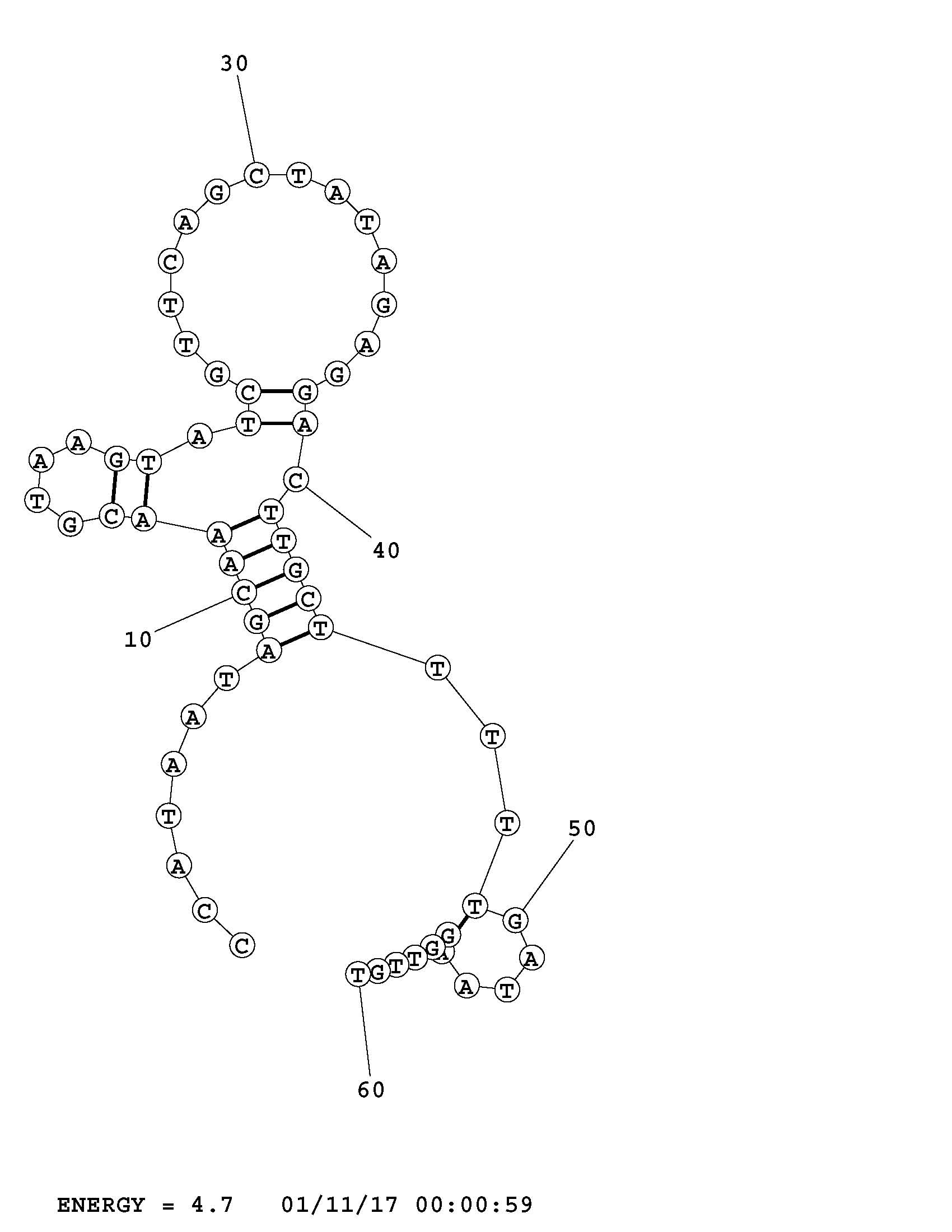

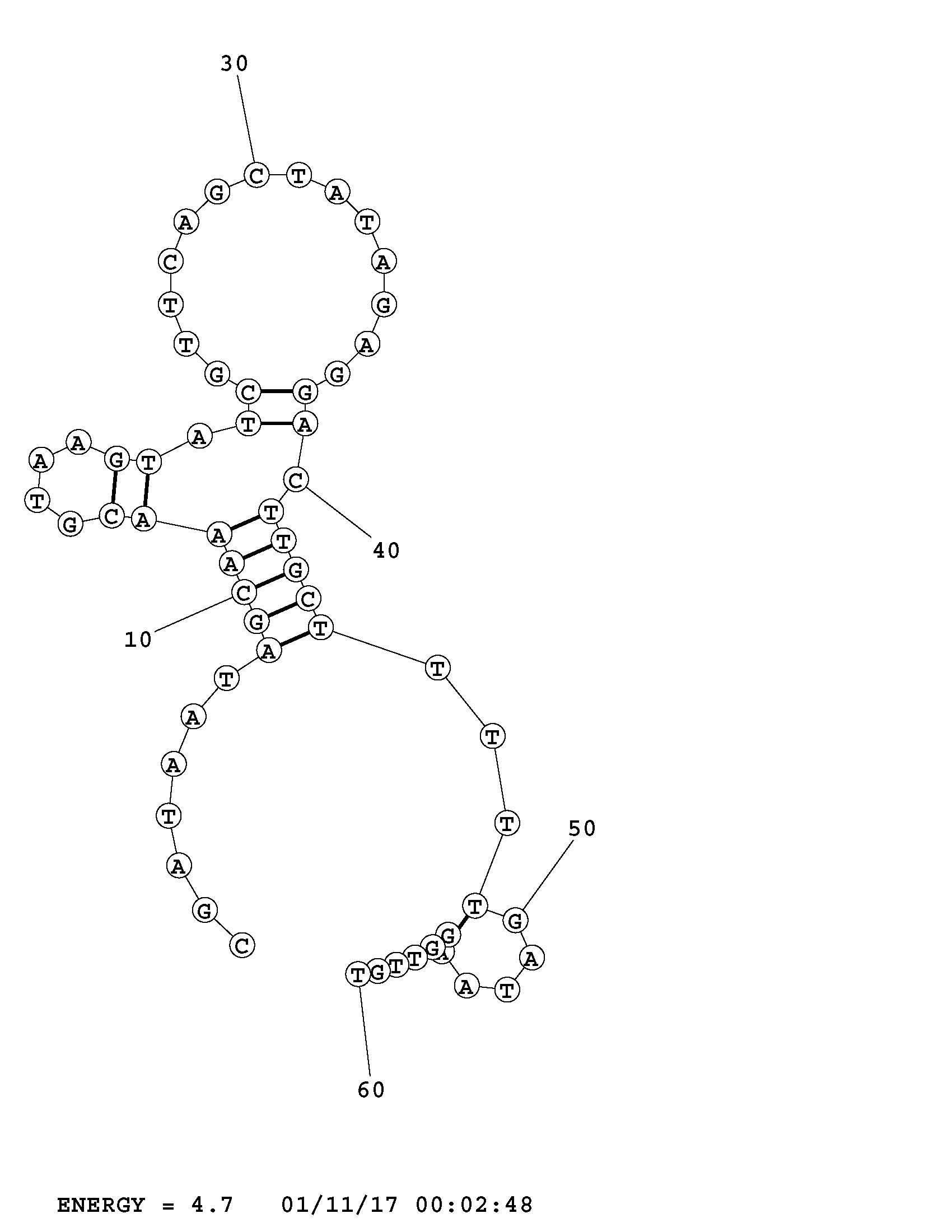


AHHF

Virulent purdue

Attenuated H


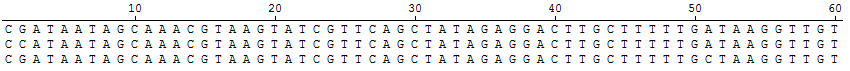

Supplement: Supplementary Figure S1 [file emi201762x3.docx]
